# Peer-to-peer support model to improve quality of life among highly vulnerable, low-income older adults in Cape Town, South Africa

**DOI:** 10.1186/s12877-019-1310-0

**Published:** 2019-10-22

**Authors:** Leon N. Geffen, Gabrielle Kelly, John N. Morris, Elizabeth P. Howard

**Affiliations:** 1 Samson Institute for Ageing Research, 234 Upper Buitenkant Street, Cape Town, 8001 South Africa; 2 Marcus Institute for Aging Research, Boston, MA USA; 3 Connell School of Nursing, Boston College, Newton, MA USA

**Keywords:** Peer-to-peer support model, Home visitors, interRAI check-up

## Abstract

**Background:**

Developing countries are experiencing rapid population ageing. Many do not have the resources or formal structures available to support the health and wellbeing of people as they age. In other contexts, the use of peer support programmes have shown favourable outcomes in terms of reducing loneliness, increasing physical activity and managing chronic disease. Such programmes have not been previously developed or tested in African countries. We piloted a peer-to-peer support model among vulnerable community-dwelling adults in a developing country (South Africa) to examine the program’s effect on wellbeing and social engagement.

**Methods:**

A pre-post, pilot design was used to evaluate targeted outcomes, including wellbeing, social support, social interaction, mood, loneliness and physical activity. A total of 212 persons, aged 60+ years and living independently in a low-income area in Cape Town were recruited and screened for eligibility by trained assessors. Participants were assessed using the interRAI CheckUp, WHO-5 Wellbeing index, and the MOS-SS 8 instruments before and after the 5-month intervention, during which they received regular visits and phone calls from trained peer volunteers. During visits volunteers administered a wellness screening, made referrals to health and social services; built friendships with clients; encouraged social engagement; promoted healthy living; and provided emotional and informational support.

**Results:**

Volunteer visits with clients significantly increased levels of self-reported wellbeing by 58%; improved emotional and informational support by 50%; decreased reports of reduced social interaction by 91%; reduced loneliness by 70%; improved mood scores represented as anxiety, depression, lack of interest or pleasure in activities, and withdrawal from activities of interest; and increased levels of physical activity from 49 to 66%.

**Discussion:**

The intervention led to demonstrable improvement in client wellbeing. Policymakers should consider integrating peer-support models into existing health programs to better address the needs of the elderly population and promote healthy ageing in resource-poor community settings. Longer-term and more rigorous studies with a control group are needed to support these findings and to investigate the potential impact of such interventions on health outcomes longitudinally.

## Background

Aging among the world populations defies borders, including developed, developing and underdeveloped regions. The dramatic increase in aging worldwide calls for health care providers and others to prepare and intervene now to best position older adults for high quality of life while providing an efficient and economical model of care to support them. For instance, the United Nations Population Division has estimated that the number of people over the age of 60 will rise from 800 million (11% of the world’s population) in 2011 to over 2 billion (22% of the world’s population) in 2050 [1]. The number of people aged 80+ is estimated to increase by 270% over the same period [1], with the greatest increase of older persons expected in low and middle income countries. In Africa, the population over age 60 will increase from 46 million in 2015 to an estimated 147 million in 2050 [2]. The South African Medical Research Council estimates that the absolute size of growth of the population over 60 in South Africa will increase by 112%, from 2.47 million in 1985 to 5.23 million in 2025 [3]. This growth presents significant challenges to resource-constrained economies with other significant competing health and social challenges [3–6].

Although few studies have been conducted, there is general agreement in the literature that access to care and health system responsiveness in developing countries is poor, and that health systems frequently fail to meet the needs of older people [4, 7]. Addressing the issue of aging within this context, the purpose of this project was to implement a peer-to-peer support model among a group of vulnerable older adults and to examine the program’s effect on the wellbeing, social engagement and physical activity of community dwelling older adults living in a community situated in a developing country (South Africa).

With advancing age comes increases in chronic conditions such as dementia, stroke, chronic obstructive pulmonary disease and diabetes mellitus [8]. Increased survival with chronic disease will account for greater disability as the population ages [8]. Even in the absence of chronic illness, lack of proactive strategies will accelerate declining physical ability as adults age. Physical limitations may lead to functional decline and the inability to care for oneself in addition to increasing risk for falls, physical activity decline, depression, loneliness and hospitalization.

For example, loneliness, defined as a subjectively experienced aversive emotional state, is related to the perception of unfulfilled, intimate and social needs [9]. Social loneliness occurs through isolation and physical loss, while emotional loneliness can occur through the absence of a reliable attachment figure such as a partner and may result in feelings of depression [9]. Studies among the 65+ age group in Britain report a prevalence of 5–16% [9]. Amongst South African adults participating in the Study of Global Ageing and Adults Health (SAGE), loneliness has been estimated to affect between 9.9, and 12.5% of those over 70 [10]. Prevalence of loneliness increases with age, due to changes in health, functional capacity and social networks [11]. Loneliness and physical loss have been associated with an increased risk of poor prognosis in late life depression [12], metabolic syndrome [13], elevated systolic blood pressure [14], mortality in men [15], and emergency hospitalisation [16].

Social wellbeing is a person’s subjective perception that life is good [17]. High social wellbeing has been shown to lower the odds of mortality and onset of disability [17, 18]. Health, physical activity, social integration, connection, relationships and social support have been frequently cited as some of the most important factors influencing self-reported wellbeing in the elderly [17, 19, 20]. A review of loneliness and social wellbeing literature reveals a paucity of information pertaining to older persons in Africa in general, and South Africa specifically. Furthermore, no intervention studies have been reported to address issues of loneliness and wellbeing.

Studies have shown that peer-to-peer programs are effective ways to provide emotional, informational and appraisal support and help alleviate transitional, chronic or acute life stressors in numerous populations [21]. Among Western populations, as well as some Asian countries [22–24], use of peer support with older adult programming has demonstrated effectiveness. A series of evidence-based programs were developed in the US to address some common geriatric syndromes. These programs address issues such as falls [25], chronic disease self-management [26, 27], chronic lower back pain [28, 29], and depression [30]. In all instances, these programs are delivered using trained older adult peers and peer volunteers who experience the same problem or threat. In Korea, Kim [22] matched trained peer supporters with community-dwelling, low-income older adults living alone. Findings showed improvements in mental health, social functioning, depression, and physical health status.

Peers have also been used to target physical activity outcomes. Dorgo, Robinson and Bader [31] found use of peer mentors produced higher retention rate in a fitness regimen that included resistance training and cardiovascular activities when compared with kinesiology student mentors. Others have found phone calls and home visits by peers improved physical activity among elders [32]. The encouragement and support from peers results in greater physical activity improvement among elders when compared with a community group intervention [33]. A study by Buman et al. revealed that peer volunteers enhanced the long term maintenance of physical activity when examined 18 months following a 16 week intervention [33].

Peer support programs present a low-cost way of supporting healthy ageing, yet few such programs have been piloted in low and middle-income countries. To address the needs of an expanding older adult population in Cape Town, South Africa, the AgeWell program was piloted in the community of Khayelitsha, a township of Cape Town in 2014. This paper reports on selected longitudinal change outcomes at the five-month follow-up.

## Methods

### Programme description

AgeWell - a community-based peer-to-peer support program - was designed as an adaptation of the successful Mothers2Mothers (M2M) program [34] for HIV positive mothers. M2M empowers and employs HIV-positive Mother Mentors to work in local communities to ensure that women and their families get the health and support they need.^1^ The AgeWell model drew on this mentor or peer supporter concept, engaging older people to provide companionship to less-able older persons living in their communities. Peer supporters or “AgeWells”, worked in pairs conducting home visits comprised of both social and wellness content. The social content was related to companionship, social support, and community engagement. Through scheduled contact, AgeWells built friendships, encouraged social engagement, provided emotional and informational support, and promoted healthy living. In addition, these peer service providers were trained to use smartphone technology, programmed with research-driven screening tools and referral algorithms to identify evolving health problems and make referrals to primary care providers and social workers**.** AgeWells collaborated with clients to draw up a “Wellness Plan” and then followed up with clients through subsequent home visits and phone calls to encourage compliance with referral recommendations and wellness plan strategies for goal achievement.

A pre-post, pilot design was used to test the feasibility of the program and evaluate targeted outcomes.

### Sample

A non-random, convenience sample of 212 persons, aged 60+ years and living independently, were recruited from Khayelitsha, a peri-urban suburb of Cape Town, and specifically, the geographic area of Mandela Park. The sample size was based on the number of people participating in older persons clubs in the area who opted into and qualified to participate in the study. Khayelitsha is a high density area with a total population of 391,749, approximately 30 km from Cape Town [35]. The population is predominantly disadvantaged black, isiXhosa speaking South Africans with limited resources and limited access to formal healthcare services. Unemployment in the area is extremely high and exceeds 38% and a quarter of the households have incomes of less than USD337. Living standards are low - over 35% of dwellings are informal shelters and many people have no piped water to their homes (65%), no flush toilets (28%), or electricity for lighting (19%) [35].

Key statistics are summarised in Table 1.

**Table 1:** Key population statistics in Khayelitsha (Source: Statistics South Africa, 2011)

Key Statistics	Khayelitsha
Total population	391,749
Elderly (65+)	1.6%
Dependency ratio	42.5
Higher education aged 20+	4.9%
Completed high school aged 20+	30.8%
Formal dwellings	44.6%
Flush toilet connected to sewerage	71.7%
Piped water inside dwelling	34.6%
Electricity for lighting	80.8%
% Households with incomes ≤ R4800 (USD 337)	24.5%
Unemployment rate	38.3%
Main language spoken	isiXhosa (90.5%)
Main racial group	Black African (98.6%)

### Recruitment and enrolment procedures

Programme staff and volunteers shared programme information with older persons visiting the sites of local care providers, community centers and community organizations. Word of mouth was also used to disseminate information about the program in each of the study areas. Potential clients were then contacted by a member of the study team by telephone for the purposes of pre-screening. Each potential client was asked:

1. “Are you under the age of 60”
2. “Do you live outside the designated geographical area?”
3. “Do you require 24 hour professional care?”

If any question was answered “Yes”, then the potential client was declined further assessment and participation in the program. The purpose of Question 3 was to exclude the most dependent older persons from the outset. The preliminary telephone screening yielded a sample of 245 for the second phase, the baseline assessment. If a client was deemed to be suitable for the program, then an appointment was scheduled for a trained assessor to visit them in their own home to determine eligibility.

The recruitment goal was a cohort of older adults who were not too frail or not too well, and thus best suited to benefit from this type of program. Those excluded for being “too frail” included those residing in an assisted living or frail care facility or requiring 24 h professional care, greater than mild cognitive impairment as calculated by the interRAI Cognitive Performance Scale > 1 [36], or functional impairment as calculated by the interRAI Functional Hierarchy Scale > 6 [37], or having an unstable medical condition plus fatigue. In addition, subjects deemed “too well” to benefit from the AgeWell program also were also excluded. This determination was based upon having *less* than 2 of the following physical or psychosocial conditions: unstable gait, mood problems, loneliness, daily pain of less than moderate or severe intensity, infrequently or never leaves home, incontinence, dyspnea, or fatigue. If the client was deemed to be too well for the program, they were encouraged to continue with their normal activities and the interview was terminated.

This secondary screening assessment was conducted by a trained assessor in the prospective client’s home using the interRAI Check-Up instrument - a comprehensive assessment tool derived from interRAI’s Health and Social Check-up and Wellness tools [38]. *interRAI CheckUp* is a geriatric assessment instrument designed to be used by non-healthcare professionals to identify losses in intrinsic capacity at the primary care level. This instrument belongs to the interRAI suite of assessment instruments, a set of third-generation comprehensive multi-dimensional instruments for use in a number of vulnerable populations (including older adults), developed by a network of health researchers from over 30 countries [39]. All instruments are built on a common set of items, as well as specialised items relevant to particular contexts and patient groups and are designed to track patients longitudinally over time and across multiple care settings. These instruments have been continuously improved and validated against other commonly used instruments for use in long-term care, acute and post-acute care, home care, palliative and community settings [40–44]. The item domains of these instruments have been shown to have good inter-rater reliability and have been adopted in across the world [45].

Two assessors, fluent in English & isiXhosa, with tertiary education, but no formal health care qualification, were given 40 h of training to use the interRAI Check-Up instrument. Training consisted of workshops, supervised assessments and mock assessments.

Data was captured onto tablet devices. Custom-written software for the Check-Up instrument allowed capture of information directly to the device, allowing inherent scales and algorithms designed by interRAI for the Check-Up to be calculated in real time. The Check-Up instrument was administered as a 3 part questionnaire.

A total of 245 assessments were completed during this second phase. Six were deemed too frail to participate and 27 deemed too well to participate, yielding a final sample of 212 adults.

### Intervention

Once a client was deemed to be eligible for the program, the remaining items of the Check-Up assessment, as well as the World Health Organisation Wellbeing Index (WHO-5) and Medical Outcomes Study Social Support Survey (MOS-SS) assessments were completed. The intent was to highlight health and social welfare conditions that could trigger the need for further investigations, improve wellbeing and reduce loneliness.

At the conclusion of the full assessment, the older adult and the assessor collaborated to develop a “Wellness Plan”. Incorporating results from the assessment, the “Wellness Plan” contained primary goals for the elder and a set of action plans or activities to pursue and support achievement of articulated goals. The plan was viewed as an individualised roadmap for each older adult, guiding them towards a higher level of wellness.

A secondary aim of this project (and primary focus of this paper) was to evaluate whether peer-to-peer companionship by Agewell visitors to other older adults could improve wellbeing and social support. Twenty-eight active and able persons, aged 60 years and older, living in the study area and belonging to a partner community-based organisation were recruited after an interview and selection process. These AgeWell Visitors (AgeWells) were given 4 weeks of training on topics such as older persons’ health, wellbeing, friendship development, smart phone operation, use of a proprietary screening instrument developed by AgeWell and referrals to social or health services according to algorithms devised by AgeWell. All 28 trainees performed well during training and were all offered positions as AgeWells. AgeWells were paid a stipend for 20 h of weekly engagement.^2^ AgeWells were paired up and allocated between 13 and 17 clients to visit according to a predefined visit schedule.

AgeWell program participants were assigned a pair of AgeWell visitors who would visit them at weekly, twice monthly or monthly intervals. The frequency of intervals was determined by the outcome of the embedded scale scores and risk indices of the InterRAI Check-Up screening instrument. Those assessed as having higher levels of disability received more frequent visits. Contact with the clients was made at more frequent intervals via telephone calls.

During visits, AgeWells administered a short 20-question wellness assessment using a smartphone. Depending on the responses, algorithms might trigger referral recommendations to medical professionals and social services providers, which were facilitated via the provision of a printed letter containing the relevant information. AgeWells then follow-up with clients through subsequent home visits and phone calls to encourage compliance with referral recommendations. Other aspects of the visits included building friendships, encouraging social engagement, providing emotional and informational support and promoting healthy living.

Enrollment to the program commenced on 20 March 2014 and completed on 7 August 2014. Services were provided from April 2014 – December 2014, thus allowing for at least 4–5 months of client exposure to the program.

### Study outcome variables

The main study outcomes, subjective wellbeing and levels of emotional and informational support were measured using the WHO-5 Wellbeing Index, the Medical Outcomes Study Social Support Survey (MOS-SS) and variables from the interRAI CheckUp instrument related to mood, loneliness and levels of social and physical activity. The CheckUp instrument was administered prior to enrolment (as part of the recruitment screening procedures described above) and at the end of the study. The WHO-5 and MOS-SS instruments were administered by an assessor at the beginning of the study.

The WHO-5 is a 5 question assessment which has been validated in a number of studies [46–48] in various populations across the world [48, 49] including numerous geriatric populations [48, 50, 51]. The total score range is 0–25, with 25 representing the highest state of wellbeing. Total scores are multiplied by 4 to obtain a percentage score. A 10% shift in score indicates a significant change in wellbeing. The Medical Outcomes Study Social Support Survey (MOS-SS) is a 19-item, self- administered social support survey [52]. The scale is considered useful for assessing changes in the levels of social support available to those who have been identified as being socially isolated. It can be used as a measure for the outcome of services or areas of work focused on reducing social isolation or increasing levels of social support [53]. Each of the scale’s four domains can be used in isolation and, for the purposes of this study, the 8-question subscale (MOS-SS 8) on emotional/ informational support was used as this best fit the profile of the AgeWell intervention. This scale has been demonstrated to be psychometrically sound, is considered universally applicable and has been used in various populations over more than two decades [53, 54].

Specific outcome variables related to psychological wellbeing, as well as physical activity and falls were measured via interRAI’s Check-up instrument. While the interRAI CheckUp includes items related to health and function, these were not the main focus of the intervention and are therefore not reported on below.

The selected outcome measures for all three instruments are summarised in Table 2 below.

**Table 2:** Descriptions of outcomes and associated measurement scale

Outcome	Measurement Scale
Well-being	WHO-5 Well-being index Scale: 0 *None of the time –* 5 *All of the time* Items: 1. I have felt cheerful and in good spirits 2. I have felt calm and relaxed 3. I have felt active and vigorous 4. I woke up feeling refreshed and rested 5. My daily life has been filled with things that interest me Total score: 0–25 (low to high wellbeing) × 4 = % score *Change in 10% or more over time is significant change in wellbeing*
Emotional and informational support	MOS-SS 8 (education and informational support scale) Scale: 0 *None of the time* – 5 *All of the time* Items 1. Someone you can count on to listen to you when you need to talk 2. Someone to give you information to help you understand a situation 3. Someone to give you good advice about a crisis 4. Someone to confide in or talk to about yourself or your problems 5. Someone whose advice you really want 6. Someone to share your most private worries and fears with 7. Someone to turn to for suggestions about how to deal with a personal problem 8. Someone who understands your problems Total score: 0–40 (low to high social support) *Score of 32 and above indicates satisfactory support*
Selected InterRAI CheckUp Variables and Associated Measurement Scales	
Mood In the last 3 days, how often have you felt… 1. Little interest or pleasure in things you normally enjoy 2. Anxious, restless or uneasy 3. Sad, depressed or hopeless	0 – do not feel this way in past 3 days 1 – often feel this way but not in past 3 days 2 – 1 to 2 days in the past 3 days 3 – daily in past 3 days
Loneliness	0 – Not lonely 1 – Lonely only in certain situations 2 – Lonely occasionally 3 – Lonely frequently 4 – Lonely daily
Participation in Activities of Long-standing Interest	0 – Never 1 – More than 30 days ago 2 – 8-30 days ago 3 – 4-7 days ago 4 – In the last 3 days
Reduced Social Interactions	0 – do not feel this way in past 3 days 1 – often feel this way but not in past 3 days 2 – 1 to 2 days in the past 3 days 3 – daily in past 3 days
Stamina – Physical Activity Level	0 – none in past 3 days 1 – less than 1 h in past 3 days 2 – 1-2 h in past 3 days 3 – 3-4 h in past 3 days 4 – more than 4 h in past 3 days
Falls	Yes/No fall in the past 90 days

### Program monitoring and evaluation

The AgeWell visitors used smartphones, allowing for paperless data collection, real-time performance management, and automatic and immediate referral triggers. An mHealth system was designed to monitor program activities and gather outcome data. Activity data was monitored to assess for timeliness and adherence to visit schedules and social/physical health screenings completed. The benefit of using e-technology to monitor much of the program implementation meant that necessary adjustments and problem solving could be expedited to ensure operational success. At the end of the programme, 3 focus groups were held with 25 AgeWells. The intention was to explore the effects of the AgeWell peer-support health model on improved wellbeing amongst AgeWells and their peer-companions and to elicit feedback on their programmatic improvement.

### Ethics approval

Ethics approval for the study was obtained from the Foundation for Professional Development-Research Ethics Committee (FPD-REC) in Johannesburg, South Africa. The trained assessors were responsible for obtaining informed written consent from all participants.

### Statistical methods

STATA was used to analyse the cleaned quantitative data collected from the WHO-5 and MOS-SS 8 instruments. Paired t-tests were used to analyse differences in wellbeing and social support scores at baselines and end-line.

SPSS was used to analyse data collected via the interRAI CheckUp instrument. For the CheckUp outcome variables, in each case the delta was established between the baseline and follow-up scores, and this value was tested with the chi-square statistic.

## Results

The mean age of participants was 69. The sample population is described in Table 3 below:

**Table 3:** Description of sample population

Mean age	69
Female	75%
Mean years education	6
Live alone	7%
Identify as primary caregiver	38%
Went to a health care provider < 1 month ago	73%
Diagnosed with diabetes	50%
High risk for loneliness	58%
No days out in the last 3 days	61%
Cannot count on friends for companionship	52
Pursue involvement in the community	61%
No exercise in last 3 days	50%

Few lived alone (7%), and 38% identified as a primary caregiver. Multi-generational households are very common in the African population and, given high unemployment and the AIDS epidemic, older people, particularly women, often take on primary care and financial care of their grandchildren using their state pensions. Despite most living with family, 58% were at high risk for loneliness, with 52% reporting that they cannot count on friends for companionship and 61% not having left the house in the past 3 days.

Most had access to healthcare and 73% had visited a primary care provider less than a month ago. This high rate of access to primary healthcare is common as primary healthcare is free in South Africa and people with chronic illnesses attend dedicated treatment clubs for regular check-ups and to receive their medication.

**Fig. 1:**
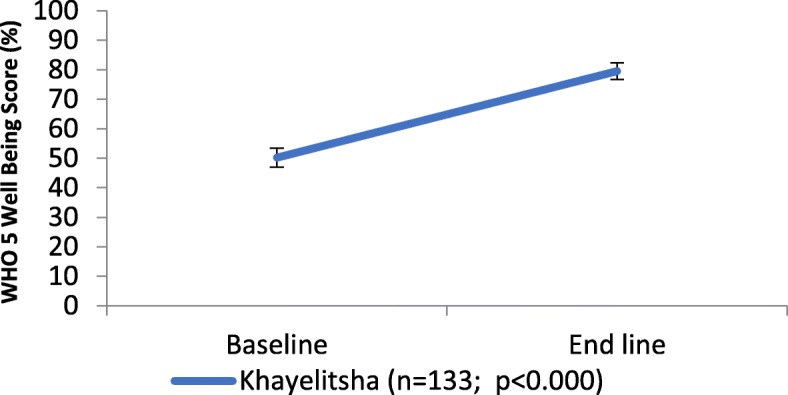
Changes in mean WHO-5 Wellbeing score in Khayelitsha program from baseline to end-line

AgeWell clients showed improvements in wellbeing with a baseline average client WHO-5 Wellbeing score of 50% rising significantly to 79%, (*p* < 0.000), an overall improvement of 58% (See Fig. 1 below). There was no association noted between changes in wellbeing score and frequency of AgeWell visits.

**Fig. 2:**
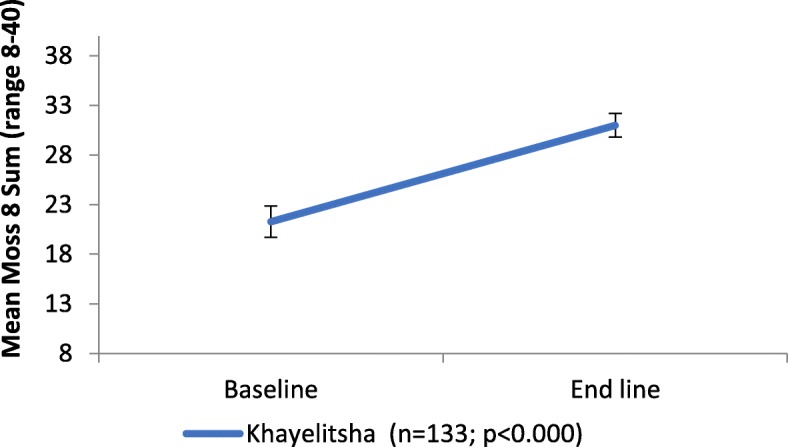
Changes in mean MOS-SS 8 Social support score in Khayelitsha program from baseline to endline

Similarly, there was a significant 50% (*p* < 0.000) increase in mean client social support scores as measured with MOS-SS 8 from baseline to endline with baseline mean score of 21 with improvement of 10 points to 31 at endline (see Fig. 2 below). Using the categorization of satisfactory social support, only 8.7% of clients scored 32 or above on the scale at baseline. By the end of the project this increased to 61.4%.

Completing a pre-post comparison using the interRAI CheckUp, all of the outcomes with the exception of falls improved significantly over the five-month observation period. Persons were less anxious, less depressed, less withdrawn, less lonely and more physically and socially active.

**Fig. 3:**
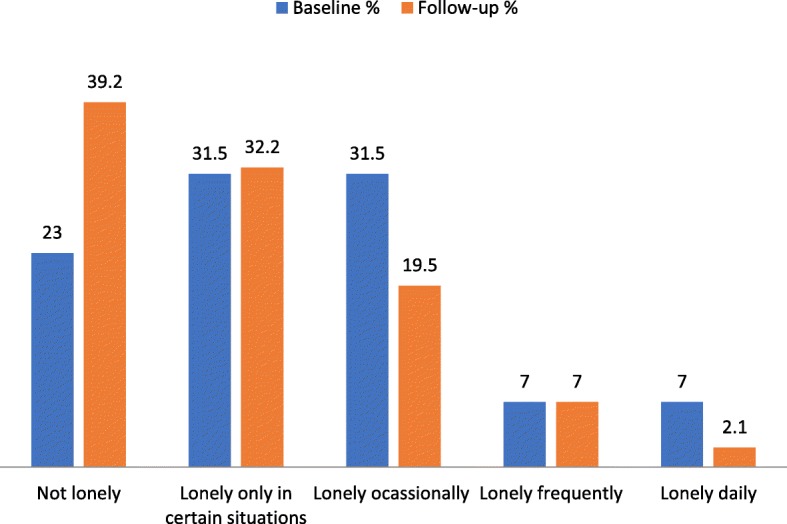
Self-Reports of loneliness at baseline and follow-up

As indicated in Fig. 3 below, only 23% of the sample reported never feeling lonely and at the end of this project, this increased to 39.2%.

**Fig. 4:**
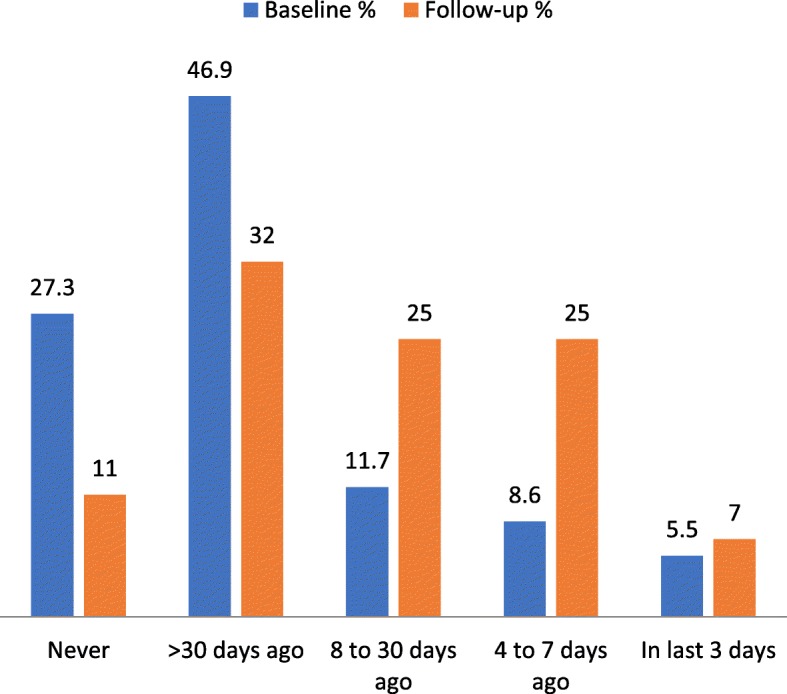
Participation in social activities of long-standing interest

This reduction in loneliness may have been associated with a decreased in the percentage of persons reporting reduced social interactions - from 41% at first assessment to 3.5% at follow-up (*p* < 0.000), possibly because of the regular visits by AgeWells. There was also a 31% increase in people reporting participation in social activities of long-standing interest over the past 30 days (Fig. 4).

Overall, the group experienced improved mood and, as Table 4 indicates, there was a significant drop in the number of people experiencing daily anxiety; feelings of sadness, depression or hopelessness; a lack of interest or pleasure in their activities; or withdrawal from activities of interest.

**Table 4:** Changes in mood scores

Item	Percentage at Baseline	Final Percentage	*P* value
Self-report anxious, restless or uneasy - daily past 3 days	16%	1%	< 0.000
Self-report little interest or **pleasure** in things you normally do - daily past 3 days	21%	1%	< 0.000
Self-report withdrawal from activities of interest - daily past 3 days	14%	1%	< 0.000
Self-report **sad,** depressed or hopeless - daily past 3 days	18%	0.7%	< 0.000

**Fig. 5:**
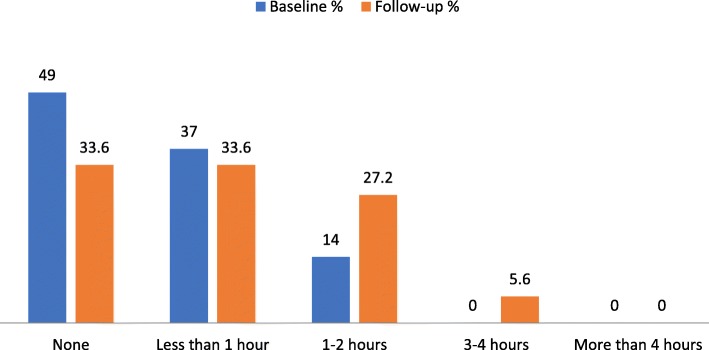
Total hours of exercise or physical activity in last 3 days

Physical activity levels were initially low, with 49% reporting no exercise or physical activity. At follow-up, there was a significant improvement with 66.4% doing some exercise or physical activity in the last 3 days. This is shown in Fig. 5 below.

While findings were not statistically significant, at follow-up 7% fewer people had experienced falls in the past 90 days (*p* = .063). There was also an increase in stamina, with the number of people experiencing no fatigue increasing from 45.5 to 54.5%. However, this finding was only significant at the .034 level.

### Programme effects on AgeWell visitors

The AgeWell programme not only benefitted those older persons visited, but also had notable empowerment effects on the AgeWell visitors themselves. Focus groups conducted with AgeWells as part of monitoring and evaluation efforts indicated that the program had an effect on their subjective psycho-social, emotional and physical wellbeing. Focus group participants indicated that since commencing the program they felt more connected to each other and their community and reported feeling less alone or isolated. The group structure and schedule of the program contributed to building authentic relationships amongst AgeWells.

AgeWells also reported improved self-esteem, and feeling a renewed sense of purpose. They were also excited to be able to learn about new technology and to make use of it and felt empowered by their new skills. AgeWells also became more aware of and more motivated to take responsibility for their own health and wellbeing and reported becoming more physically active.

Receiving a small salary for their work gave both monetary value to their work and allowed them to become economically active. This had a considerable effect on the welfare of their family and their self-esteem and alleviated some of the disempowering effects of poverty.

## Discussion

The results from the pilot study demonstrated that a simple, low-cost intervention of visiting older persons, identified as lonely, can significantly increase self-reported wellbeing, levels of emotional and informational support, levels of social interaction, reduce loneliness, improve mood scores and increase levels of physical activity.

Home visitations and telephone calls created opportunities for social contact critical for reducing social loneliness. Given their shared experience of ageing, AgeWells were able to genuinely understand and empathise with the challenges faced by clients. This helped peer supporters to genuinely connect with clients and build trust. These relationships also acted as a motivating factor that provided participants with a common activity to look forward to. In some cases, genuine friendships emerged between AgeWell visitors and their clients, reducing emotional loneliness.

Given the well-established relationship between loneliness, psycho-social wellbeing and morbidity and mortality [18], peer supporters are well-positioned to effect preventive and promotive health in this population, alleviating downstream costs incurred from hospitalization and institutionalization.

Peer supporters such as AgeWells also have the potential to form an important link between community-dwelling older persons and health and social services.

While the study did not demonstrate improved health status, it is likely that continued intervention with sustained improvements in quality of life, mood and physical activity and reduction in loneliness may have long-term positive effects on general health and physical function.

In terms of project implementation, there was high demand for the intervention from clients, driven in part by the close-knit community, involvement of community leaders (some of whom were AgeWell visitors), the desire for social engagement and no reluctance to accept safety net services. Although client refusals were not tracked, overall client attrition from the programme was 10%, with relocation accounting for 35% of programme dropout. Challenges faced included political unrest, which could interfere with enrolment and visits and linking clients to health and social services given the overburdened and under-resourced health and social systems and difficulty in tracking referrals. While AgeWell visitors were enthusiastic, training older persons on administering the AgeWell wellness screening tool was challenging and their struggles using the tool could distract from the social visit.

### Limitations of the study and generalizability

It is possible that some of the improvements seen in the study population were due to initial excitement and expectations at being included in a program that values a largely neglected and marginalized group of people. It would be important to see if these gains could be meaningfully sustained over longer periods of time through further study. Furthermore, the study is based on a small sample of participants and, given that there was no control intervention, it is not possible to establish whether it was peer-to-peer support produced the improvements in psychosocial outcomes observed in the results or other factors internal or external to the programme. The CheckUp, MOS-SS and WHO-5 instruments are all self-report instruments, which may introduce biases in terms of social desirability and may be affected by participant feelings at the time of the assessment or issues around recall. However, the study does indicate that social interventions to improve the wellbeing of older persons in LMICs are worth investigating through further longer-term and more rigorous studies, such as a randomized control trial that engages with both psychosocial and health outcomes should be undertaken.

## Conclusions and implications

Innovative and effective strategies need to be developed to address the impending health and social welfare burden of ageing in resource poor settings. The AgeWell program has demonstrated improvement in client wellbeing and social support in a low-income community. Older persons, familiar with the challenges faced by the elderly are well-suited to identifying and responding the needs of older persons and providing social support to prevent loneliness and depression.

Policymakers should consider integrating peer support models into existing health programs to better address the needs of the elderly population and promote healthy ageing in community settings. Peer interaction can reduce social and possibly also emotional loneliness, promote health and wellbeing, and allow older persons to remain at home longer. As Steptoe et al. [55] argue, “health-care systems should be concerned not only with illness and disability, but also with supporting methods to improve positive psychological states. Through prevention measures for early intervention and disease management, this type of intervention also has the potential to positively affect health outcomes for the elderly.

## Data Availability

The datasets used and/or analysed during the current study are available from the corresponding author on reasonable request.

## Abbreviations

M2M: Mothers2Mothers
MOS-SS: Medical Outcomes Study Social Support Survey
WHO-5: World Health Organisation Well-being Index

## Footnotes

1 M2M provided technical, infrastructural, legal and human resource support for all administrative aspects of the project.

2 Given high rates of unemployment and poverty in South Africa, stipend-paid volunteering is very common.
